# Concrete Tank Failure as the Result of Implementing Wrong Boundary Conditions for Wall Support—Case Study

**DOI:** 10.3390/ma14102474

**Published:** 2021-05-11

**Authors:** Łukasz Drobiec, Jan Gierczak, Rajmund Ignatowicz, Piotr Kozioł, Tomasz Nowak

**Affiliations:** 1 Faculty of Civil Engineering, Silesian University of Technology, Akademicka 5, 44-100 Gliwice, Poland; 2 Faculty of Civil Engineering, Wroclaw University of Science and Technology, Wybrzeze Wyspianskiego 27, 50-370 Wroclaw, Poland; jan.gierczak@pwr.edu.pl (J.G.); rajmund.ignatowicz@pwr.edu.pl (R.I.); piotr.koziol@pwr.edu.pl (P.K.); tomasz.nowak@pwr.edu.pl (T.N.)

**Keywords:** concrete structures, concrete rectangular tank, post-installed rebar, chemical bond, pull-out cantilever–wall connection, non-destructive testing, finite element analysis

## Abstract

Damage to large reinforced concrete structures is rarely due to design errors. Sometimes, however, a small error can lead to major damage and costly repairs. The article describes the damage, the results of non-destructive and destructive tests, the results of numerical calculations, and the method of repairing a reinforced concrete tank in a sewage treatment plant. The failure was caused by applying the wrong boundary conditions to the reinforced concrete wall support inside an existing biological reactor. During leak testing, one of the new walls cracked and was displaced, which resulted in the tank leaking. An inspection of wall damage and displacement was carried out on termination of the leak testing and while the tank was draining. The causes of the failure were determined based on the inventory information and numerical simulations. Both non-destructive tests of reinforcement and concrete and destructive tests of concrete were carried out. The concrete class of the foundation slab was determined based on a compression test of sample cores obtained from drilling. The aim of the non-destructive tests was to indicate the location and diameter of reinforcement in the damaged wall using electromagnetic and radar methods, as well as the location of internal defects using ultrasonic and radar methods. It was found out that the failure was a result of an incorrect determination of the anchoring length of the reinforcement. Based on the analysis, a plan to repair the damaged wall was formulated and then successfully implemented. In the article the authors proposed the IVD (identification-verification-design) scheme to make the design easier in similar cases.

## 1. Introduction

Structural failure and collapses are usually the consequence of various factors, such as the technology applied during operation [1,2], installation technologies [3,4], errors in construction [5], or changes in settling conditions at the exploitation stage [6]. Building collapse or failure can also be the result of errors made at the design stage, for example, errors in determining the static scheme [7]. The case described in this paper involves a situation where erroneous parameters were adopted for the reinforced concrete support wall in a tank under renovation.

The use of bar anchoring techniques has been well known for many years and is used in extensions to existing buildings. Manufacturers of anchorage systems provide calculation programs for designers based on their research and standard regulations. The recommendations of the manufacturers of anchoring systems focus on the quality of the hole in the concrete, assuming at the same time that it will be made perfectly, will not be wet, and will be cleaned. Current scientific and materials research focuses on the local failure mechanism, leaving aside the global load-bearing capacity of the element to which the new element is to be fixed. Despite the efforts of modern researchers on the influence of various adverse conditions such as anchoring of bars in a wet hole [8] and the influence of the thickness of the adhesive on the bond with the concrete [9], one cannot expect an evaluation of these factors on the load-bearing capacity in the form of standard provisions.

**Figure 1:**
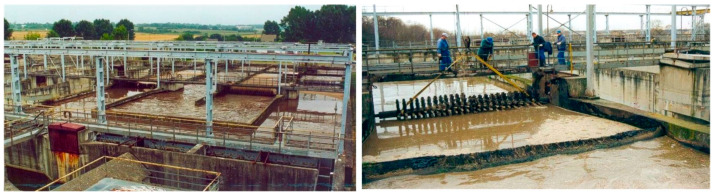
Archival photographs of the biological reactor in the 1990s.

**Figure 2:**
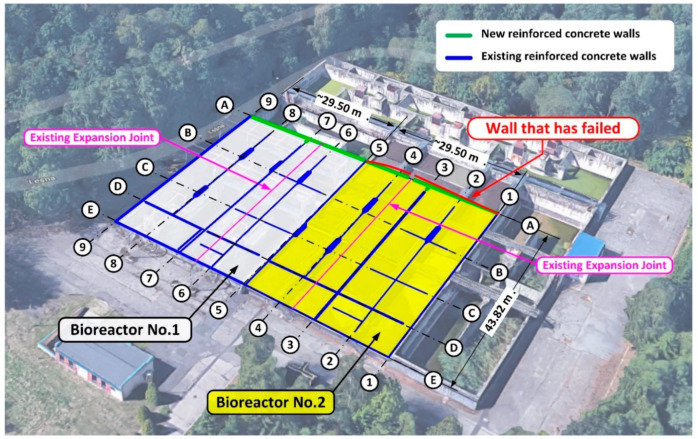
Scheme of renovation of the existing biological reactor. In the figure, the wall which failed during the leak testing is denoted on the A/1–5 axis, [m], (Google Map).

The biological reactor was brought into operation in 1989 (Figure 1). However, the innovation of the applied technology required a reduction in the surface area and in the capacity of the existing tank. In the second half of 2018, renovation works and technological upgrades of the biological reactor in the waste treatment plant began. It was decided to infill the remaining unused space to the level of the existing terrain with building materials (Figure 2).

The reduction of the tank capacity allowed the structure to be optimized. Optimization covered both the reduction of the amount of the material needed and also simplification in the installation process. Then, the full anchorage of the planned wall to the existing foundation slab of the reactor was designed.

As far as anchoring the wall to the existing foundation is concerned, the rebars were fixed to a concrete floor with an adhesive [10,11]. It is a popular, easy to use, and commonly applied method of joining together old and new structures [12,13,14,15,16,17]. Although fixing anchors and rebars with adhesive into concrete is nothing new, the methods of doing it have been being refined for the last 50 years. Nevertheless, the current knowledge combined with the technical progress enables joining new and existing concrete elements or structures in such a way that the load-bearing capacity of the join is equivalent to a monolithic structure [18].

Post-installed reinforcing bars required using so-called chemical bonds. Usually, this means applying a chemical system comprising two components: a resin (most commonly epoxy-acrylic, polyester or epoxy) and a hardener, which, when mixed together, harden in an appropriate adhesion to existing concrete. The joining (anchoring) of the rebar in the concrete floor is achieved by means of a mechanical interlock between the adhesive cement and the ribs of the rebar, as well as the adhesive forces and micro-interlock due to the roughness of the borehole surface at the adhesive-concrete interface. The resistance of the rebar join [18] may result from the following mechanisms: pulling the bar out of the resin, pulling the resin out of the drilled hole, scratching the concrete, pulling out the bar, resin, and concrete cone, and breaking the bar itself.

The first type of failure, namely a pull-out of the rebar from the resin, is not common. The production of chemical bonds is now strictly controlled, and the products undergo detailed testing in the process of preparing technical specifications [19] and due to the obligation of the European Technical Assessment standards, which are required in most European countries [20]. Resins and polyurethane glues are designed to ensure that the rebar will not be pulled out of a correctly applied anchor. Thus, it can be assumed that failure through a pull-out of a rebar is in principle only possible when there are flaws in the anchor material. In practice, the second type of failure occurs when the anchoring of the rebar is carried out incorrectly, especially when it comes to preparing the opening. For instance, when the dust leftover from the drilling is incorrectly cleaned, the resin or glue will not join with the concrete and this type of failure may occur. The third type of failure involves pulling out the concrete cone, which occurs with low concrete strength or too shallow an application of the adhesive to fix the rebar. This last type of failure turned out to be of key importance to explain the failure that occurred in the case presented below.

## 2. Description of the Structure and Design Solutions

In the second half of 2018, renovation work and technological upgrade of the biological reactor in the waste treatment plant began. Reconstruction of the biological reactors involved a partial change of the internal wall arrangement and also a reduction of the reactor capacity by installing new walls along the A axis (Figure 2).

**Figure 3:**
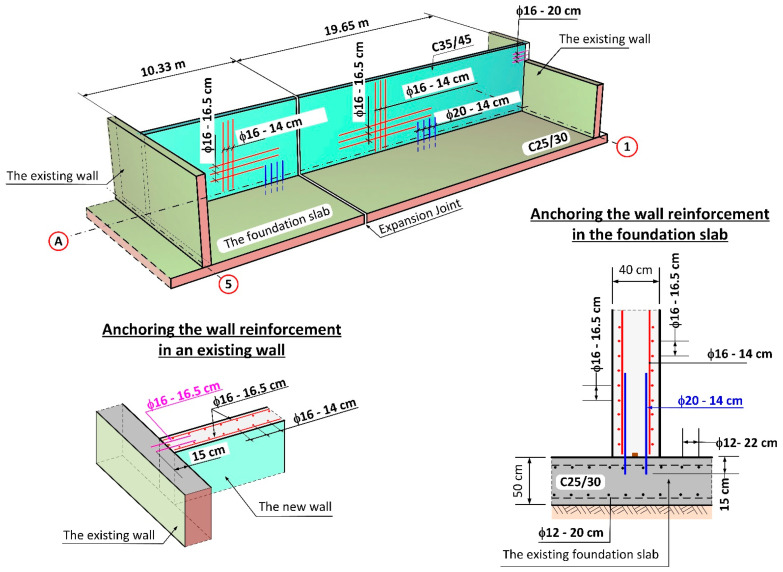
Scheme of the new wall structure on the A/1–5 axis according to the project design.

The concrete class for walls was assumed as C35/45, and reinforcement as B500SP steel in the form of double-sided steel mesh with ϕ 16 mm and ϕ 20 mm rebars. The thickness of the wall on the A axis was 40 cm, whereas the wall height from the upside surface of the foundation slab of the reactor was 5.0 m. The project design involved fixing the planned wall into the foundation slab of the reactor. The rebars were fixed to the foundation slab with adhesive one of the systems available on the market [19,20]. The design of the anchoring in the foundation slab involved vertical rebars with a diameter of ϕ 20 mm at a depth of 25 cm, spaced out at 14 cm intervals with a localized denser arrangement at 10 cm intervals (near expansion joints). Strengthening of the main vertical walls along the A axis involved ϕ 16 mm rebars spaced at 14 cm intervals, whereas horizontal strengthening was spaced at 16.5 cm intervals. Anchoring of the new wall in place of the existing walls of the old reactor on axes 1–5 was realized using horizontal rebars with a diameter of ϕ 16 mm embedded at a depth of 15 cm and with a spacing of 16.5 cm. The anchoring of the 20 cm thick internal walls was planned in a similar way. A scheme of the wall structure on the A/1–5 axis according to project design is presented in Figure 3.

**Figure 4:**
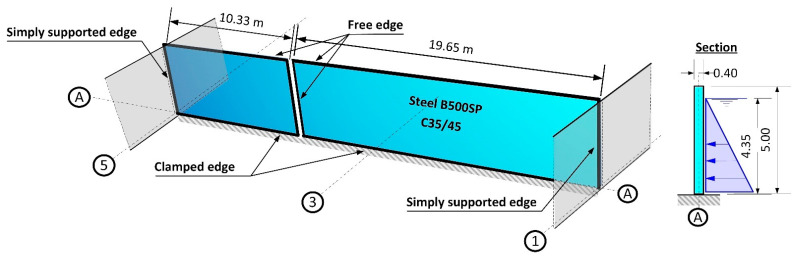
Static scheme adopted in the project design for walls on the A axis for the leak testing of the biological reactor.

The project design engineer did not calculate the length required for the anchoring in the foundation slab, which must have been taken into consideration when fixing rebars with adhesive. The design engineer adopted a nominal depth without verifying whether it was sufficient in relation to the possible failure of the rebar. Based on the catalogue of the anchor manufacturer [19], the engineer adopted a depth for fixing of the rebars as equal to 25 cm for ϕ 20 (B500SP) rebars and did not apply any calculations for verification purposes. The rebars for attaching the edges of the vertical wall in the place of the old walls were selected in a similar way. A static scheme with loadings adopted by the design engineer for the leak test is presented in Figure 4.

## 3. Leak Testing and Wall Failure

**Figure 5:**
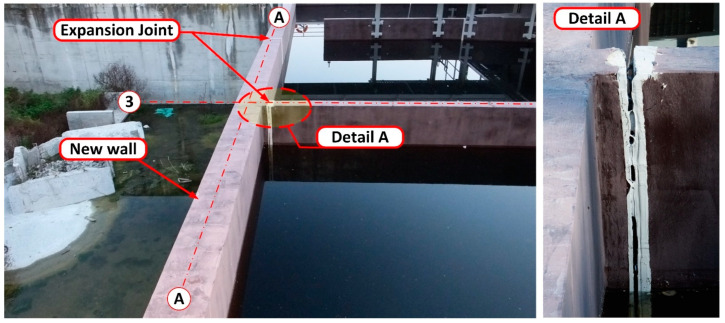
Leak testing of the biological reactor. View of the wall on the A/1–5 axis. Failure of the expansion joint at the interface between the vertical wall on axis 3 is visible. The first 12 h of leak testing.

Completion of the building work on reactor 2 was planned for mid-September 2018. As part of this process, the reactor vessel was filled with water to a level of 4.35 m above the bottom and leak testing was initiated—Figure 5.

**Figure 6:**
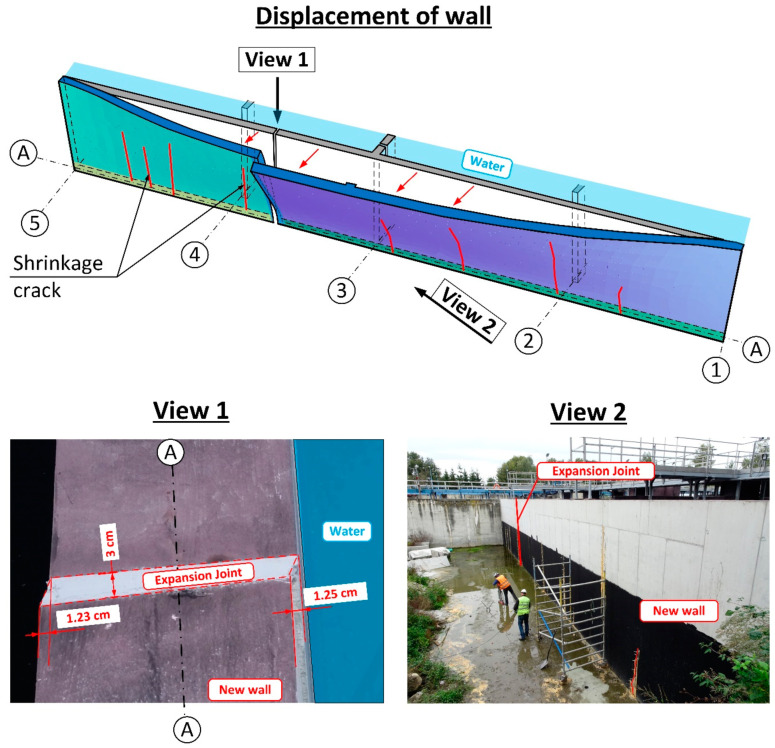
Wall displacement on Axis A following termination of leak testing. Destruction of the seals of the expansion joint (dilation) of the wall on the 3 axes.

During the leak testing, leaks appeared showing the location of shrinkage cracks [17]. Shrinkage cracks appeared in 8 places along the whole length of the wall. The cracks started at the base of the wall and reached a height of no more than 2.50 m. A progressive wall displacement was also noted, as well as a small deformation, which alerted the building inspectors to the danger of the wall collapsing along its whole length. Wall deformation was especially visible where the leakage was occurring at the interface with the expansion joint between the wall on the A axis, and the wall perpendicular to it on the 3 axes. After 24 h, further displacement of the new wall was observed and the crack at the interface of the vertical wall had increased to a value of about 7 cm. At this point, the deflection and deformation of the wall from the plane were clearly visible to the naked eye—Figure 6. For this reason, it was decided to immediately terminate the leak testing, and to drain the reactor to a level of 1.0 m above the tank bottom.

Once the tank was completely drained, it was determined that the wall deflection was partly reduced to an average about 1.0–2.5 cm. On the inside of the tank, a crack was observed at the base of the wall at the interface between the old and new concrete of ~0.7 cm indicates that joining the wall with the foundation slab of the existing biological reactor was not completed.

## 4. Destructive and Non-Destructive Tests

**Figure 7:**
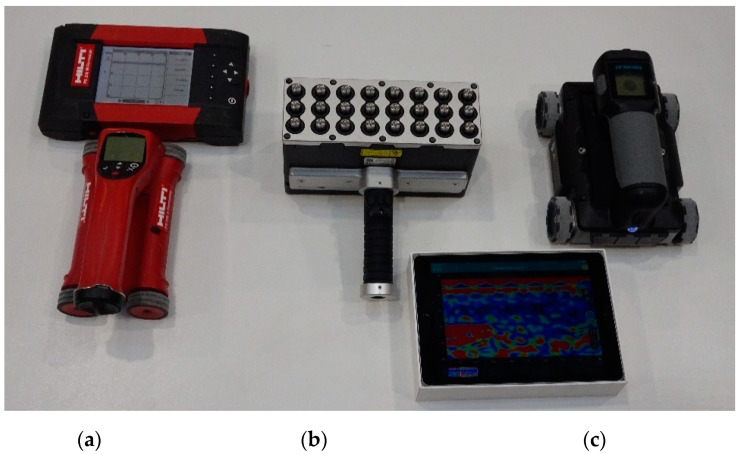
Instruments used in the tests, (**a**) electromagnetic scanner, (**b**) ultrasonic tomography, (**c**) radar scanner.

Both non-destructive tests of reinforcement and concrete, and destructive tests of concrete were carried out. The aim of the NDT (non-destructive testing) was to indicate the location and diameter of reinforcement in the damaged wall using electromagnetic and radar methods, as well as the location of internal defects using ultrasonic and radar methods. Devices used in the tests are presented in Figure 7 and described below.

**Figure 8:**
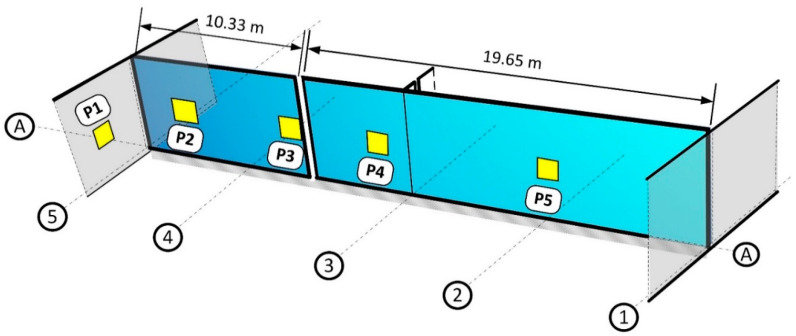
Places where electromagnetic, radar and ultrasonic tests were performed.

The PS 200 electromagnetic scanner was used to determine the location and diameter of reinforcement (Figure 7a). The sensor area was equipped with a circumferential transmitting coil and seven receiving coils. The electric current induced in the receiving coils was analysed. The detection range of the device is up to 10 cm. Five scans of reinforcement of 0.36 m^2^ area each were made on the tested wall. The location of the tested areas is shown in Figure 8.

The Pundit 250 Array ultrasonic tomography for concrete was also used during tests (Figure 7b). The instrument contains 3 rows of 8 transducers operating on a frequency 50 kHz. Each pulse-echo transducer transmitted an ultrasonic signal and echoes were received by the remaining transducers. Each transducer transmitted its own signal one by one with a delay of 8–200 ms. Complete measurement in one row consisted of 28 scans. The instrument was used to locate the reinforcement and detect internal defects in the concrete structure. Five, 80 cm long linear scans were made in the middle of the tested area of the PS 200 scanner (Hilti, Schaan, Liechtenstein).

For radar tests, we used the GPR Live System with antennas generating measurement signal of stepwise variable frequency within the range of 0.2–4.0 GHz (Figure 7c). In the case of the tested elements, the upper limit of obtained frequencies was particularly important. The maximum thickness of the tested elements was 34 cm. Therefore, the scans covered almost the entire wall thickness. The device uses the time-of-flight method (TOFM), conducting a simultaneous recording of the received signal and the movement of the measuring instrument. The GPR Live System was used to locate the reinforcement and detect internal defects of the concrete structure. Five scans (of 0.36 m^2^) were made on the tested wall. The scans were made in the same places as those of the PS 200 scanner.

**Figure 9:**
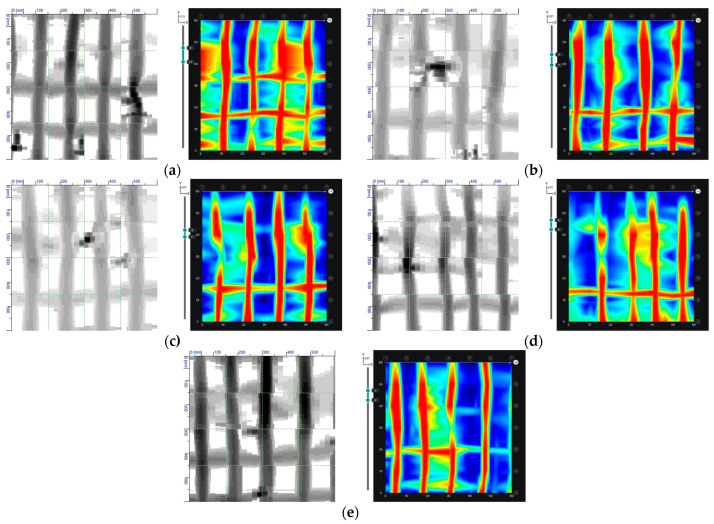
A comparison of the results of the reinforcement detection using electromagnetic and radar methods: (**a**) P1, (**b**) P2, (**c**) P3, (**d**) P4, (**e**) P5.

The conducted NDT showed the compliance of the reinforcement with the project in terms of quantity and location of rebars. However, the concrete cover of the bars was found to be slightly larger than the designed one. The reinforcement was at an average depth of 55 mm, with a standard deviation of 6 mm. This fact was considered in the design by taking the average concrete cover. A comparison of the results of the reinforcement detection using electromagnetic and radar methods is presented in Figure 9. It was found that the diameters and position of the reinforcing bars are in accordance with the design.

**Figure 10:**
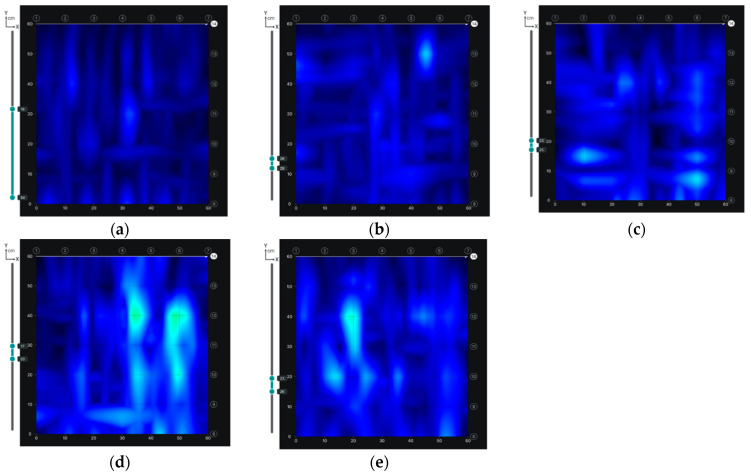
Testing of the location of internal defects in concrete using the radar method in measuring points: (**a**) P1, (**b**) P2, (**c**) P3, (**d**) P4, (**e**) P5.

**Figure 11:**
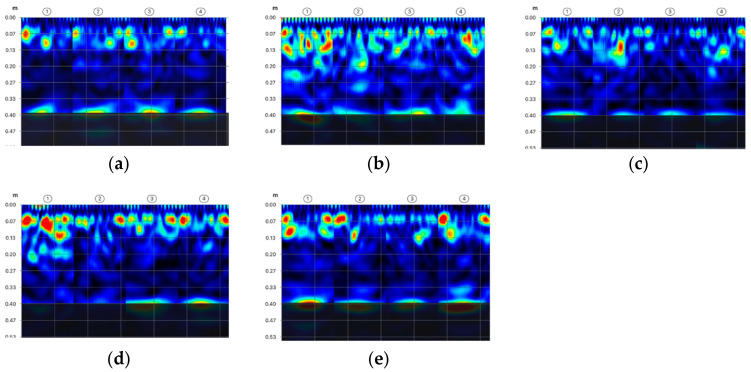
Testing of the location of internal defects in concrete using the ultrasonic method in measuring points: (**a**) P1, (**b**) P2, (**c**) P3, (**d**) P4, (**e**) P5.

Based on the scans made using the radar method, it was also possible to determine the occurrence of internal defects in the concrete. Images of radar scans at depths behind the reinforcement zone are shown in Figure 10. Internal defects of the concrete are light blue in colour. Only in the first scan, no internal defects were found (Figure 10a). The scans made in P2 and P3 have small defects, while in the P4 and P5 scans larger defects of about 30 × 30 mm were diagnosed. Similar results were obtained using the ultrasound method (Figure 11). The first scan shows only the reinforcement in cross-section (Figure 11a). In the scans taken in subsequent places, there are already areas of discontinuity in the concrete structure. The experience of the authors [21,22] shows that the applied ultrasound device gives more accurate results. On the ultrasound scans, the areas of internal defects are slightly smaller. It can be assumed that the dimension of internal defects in the analysed wall does not exceed 20 mm.

**Figure 12:**
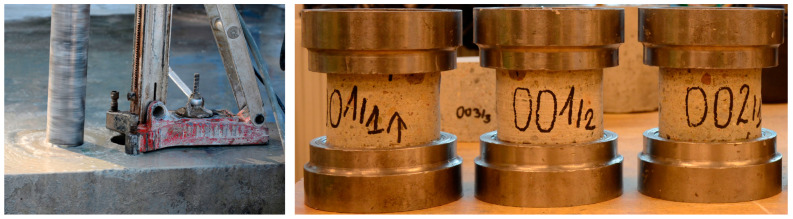
Testing for strength: core drilling and concrete specimens prepared for testing.

The effectiveness in anchoring the rebars is directly related to the quality of the concrete bases, in which they are to be embedded. For this reason, compression tests were carried out for the concrete making up the existing foundation slab to determine the concrete class. The concrete class of the foundation slab was determined on the basis of a compression test of sample cores with a diameter of ϕ 100 mm and a height of 100 mm obtained from drilling (Figure 12), in accordance with the methodology presented in [23,24,25,26]. The results of these tests are presented in Table 1. When determining the strength of concrete, the Neville formula [27] was taken into account, according to which the strength of a cube sample with a side of 150 mm equals 98% of the strength of a cylindrical sample with a height and diameter of 100 mm.

## 5. Numerical Analysis of the Wall Failure

A static calculation was carried out using the finite elements method of the SOFiSTiK system [29]. The aim was to determine the causes and progress of the failure and to identify the mechanism that initiated it. Advanced numerical simulation [29], aimed at representing realistic boundary conditions, was used. The numerical models of the damaged wall were created based on the researchers’ experience [5] and guidance in the literature [30].

**Figure 13:**
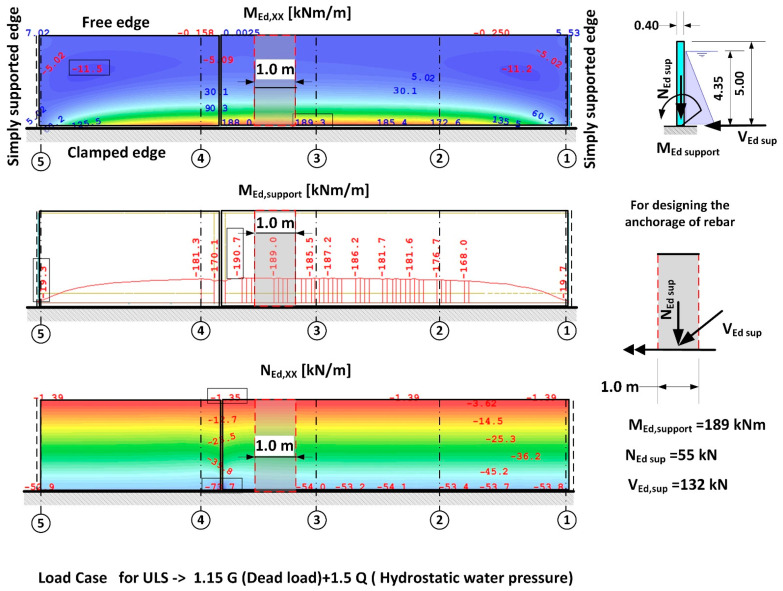
Static scheme of the wall for determining the reactions of the supports for verifying the load bearing capacity of the rebars in the foundation slab.

As a first step, according to [31,32,33], the design correctness of the strengthening of the reinforced concrete wall assuming a rigid fix to the foundation slab was analysed. It was determined that the design strength was correct in relation to the flexural behaviour, and the wall did not indicate any excessive deformation. Obtained support reactions were used for verifying the load-bearing capacity of the anchorage of the wall connecting rebars on the A axis with the existing foundation slab. The static scheme and the values of the internal forces assumed for calibrating anchorage in the foundation slab are presented in Figure 13.

The next step of the verification involved checking on the correctness of the design length of the anchorage in the foundation slab of the existing reactor. The calculations were carried out for B500SP steel rebars (*f*_yk_ = 500 MPa), which according to the project design were to be anchored at a depth of 25 cm in the foundation slab using the Fischer company injection system [20,34]. According to the acceptance protocols, the fixing of the rebars and preparation of openings were carried out in accordance with the instructions of the manufacturer under the supervision of the investor. For this reason, incorrect preparation of the openings for post-installed reinforcing bars was not considered.

**Figure 14:**
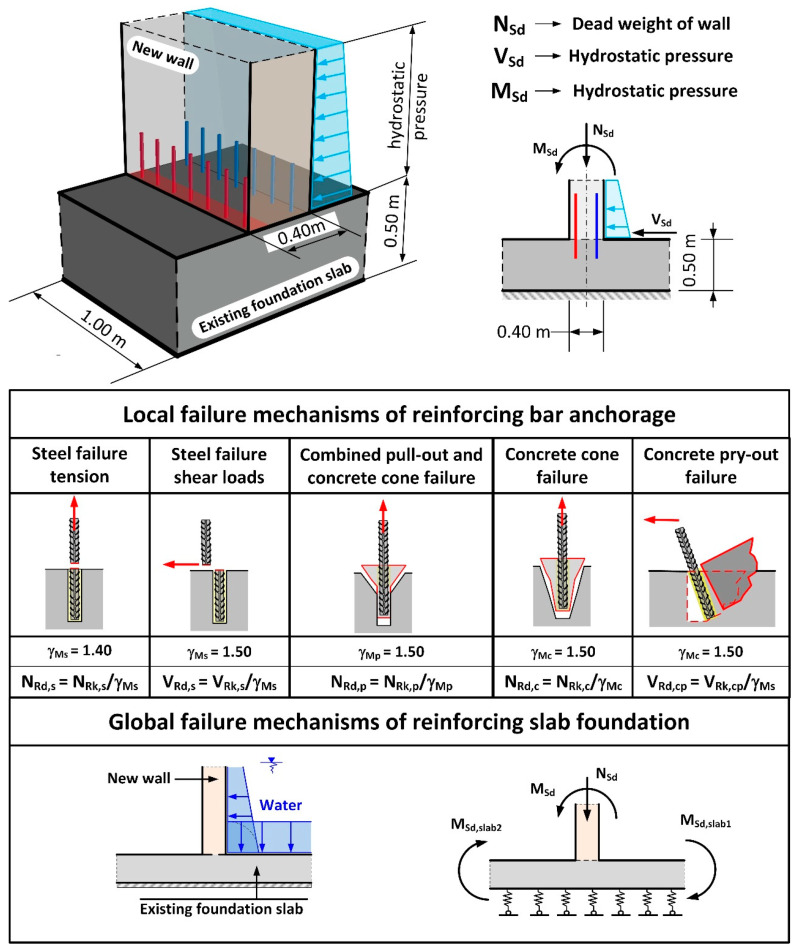
Static diagram of anchoring the wall in the foundation slab of the existing tank. Local failure mechanisms and global failure mechanism considered in the analysis.

The results of the calculations carried out in accordance with [18,20,33] indicated (Table 2) that the anchorage depth in the foundation slab was too small and that the pull-out of the anchored part of the rebars on the side with water pressure was inevitable, due to a formation of a quasi-articulated joint. The calculation of the anchorage resistance was carried out based on DD CEN-TS 1992-4-5:2009 (Design of Fastenings for Use in Concrete, Part 4–5, Post-installed fasteners-Chemical systems [18]) and all markings were taken in accordance with the standard. The results of the calculations and the static scheme adopted for the purposes of calculating the loading capacity of the anchorage applied in the project design are presented in Table 2, Table 3 and Table 4, and in Figure 14. The results indicate that the most likely failure mechanism was a pull-out of the concrete cone as the utilization ratio for this failure mechanism was the highest and amounted to 254%. Additionally, calculations were carried out to determine the minimum strengthening required, taking into consideration concrete shrinkage, in accordance with [31,35,36]. These indicated that the amount of horizontal strengthening provided was adequate. For this reason, the diagonal cracks which appeared (Figure 6) were most likely the result of negligence in curing the concrete.

The authors sought to answer the question of whether it was possible to have implemented a full anchorage in the existing foundation slab of the tank. For the purposes of the analysis in this case, it was necessary to assume that the maximum anchorage depth should be no greater than 40 cm. It must be noted that the existing foundation is water tight. With a 50 cm foundation slab thickness, there still remains 10 cm of undisturbed concrete structure. Failure mechanisms of the rebars in the wall-foundation slab connections were presented in Figure 14. These mechanisms concern both the bearing capacity of the reinforcement (local mechanisms) and the bearing capacity of the foundation slab (global mechanisms).

**Figure 15:**
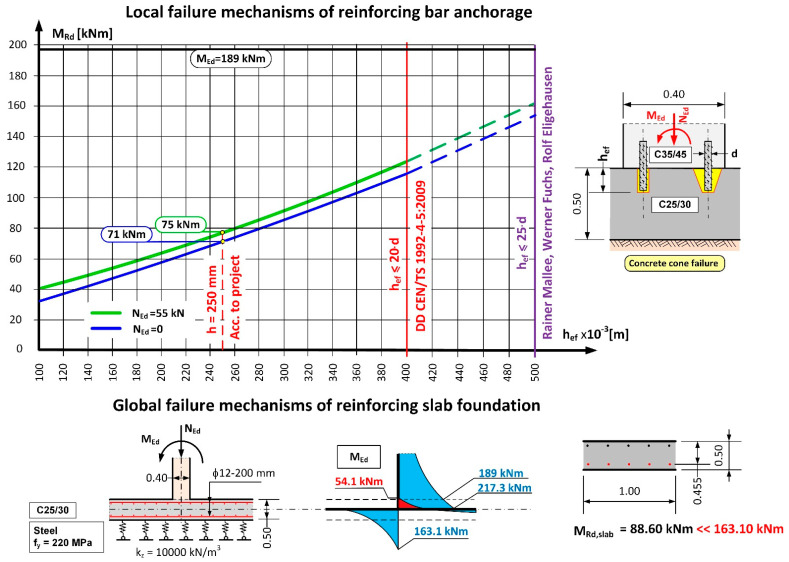
Anchorage capacity of the wall and foundation slab connection from the condition of the anchorage length of the bars [17,18] and the bending resistance of the foundation.

To answer the question posed earlier, the value for the anchoring moment in the foundation slab was set as a function of the anchorage depth for a ϕ 20 mm rebar in accordance with [18,34]. In the calculations, the two most significant failure mechanisms were considered: concrete cone failure and combined pull-out of the rebar with concrete cone failure. The results of the calculations are presented in the form of a graph in Figure 15. Analysis of the calculation results in relation to the value of the calculated anchorage moments, which were measured during the leak test, indicates that there was no possibility of achieving a complete anchorage of the wall in a slab which is 50 cm thick. For the anchorage depth adopted in the project design (25 cm), the maximum possible anchorage moment, taking into account a favourable impact of the compression force from the self-weight of the wall (N_Ed_ = 24 kN/m^3^ × 0.40 m × 5.00 m × 1.00 m × 1.15 = 55 kN) amounts to 75 kNm. On the other hand, the calculated anchorage moment for the wall in the foundation plate obtained from the static calculations (Figure 13) amounts to 189 kNm, which is equivalent to loading in the order of ~255%. It should be noted that the maximum anchorage length of rebars according to [18] is 20d, which makes it impossible to achieve the required bending resistance M_Rd_. The key to solving this problem is to use deep anchorage and check the connection with the strut and Tie (S-T) method for reinforced concrete structures. The second important criterion is sufficient bearing capacity of the foundation slab due to bending and cracking. In the case under consideration, the bearing capacity of the foundation slab is twice as low.

Therefore, the design concept adopted as a basis for realising the wall was destined for failure from the start as there was no possibility of achieving a completely effective anchorage in the foundation.

## 6. Repair Design

**Figure 16:**
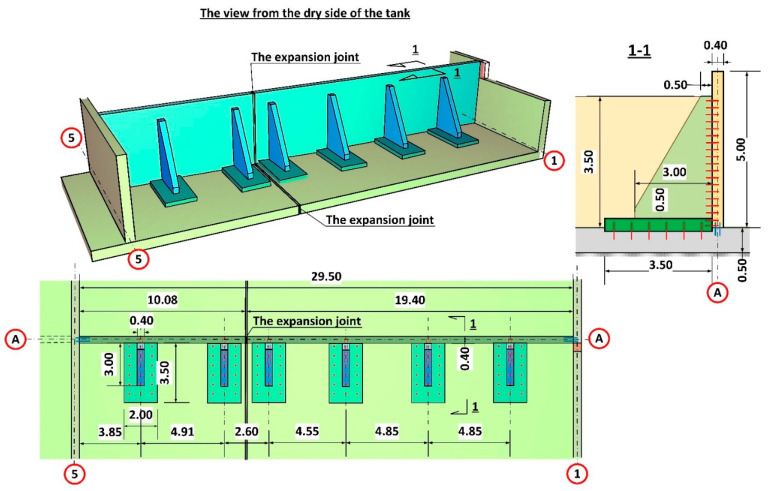
Support concept for the damaged wall in reactor no. 2.

Both numerical analyses carried out in SOFiSTiK [29] and inspection of the construction site indicated that the designed solution should be changed to enable strengthening without removal of the damaged wall. The new solution has been applied in reactor no 1 on the A/5–9 axis in order to avoid a similar failure. It was decided that buttresses, in the form of reinforced concrete pilasters, would be used to provide direct stiffening and support for the damaged wall, especially with consideration of the loading on the damaged anchor’s reduction. The final design concept for strengthening, which was approved by the investor and the design engineer, is presented in Figure 16.

The numerical model using the shell element implemented in the program ASE is a surface element [28]. The individual elements are plane and they lie in each case in a plane, the normal of which is generated through the vector product ((X3–X1)·(X2–X4)) of the diagonals. The deviation of the element’s plane from the nodes is taken into consideration using additional eccentricities. Therefore the element also works correctly with twisted geometry. The local coordinate system is oriented in such a way that the *z*-axis is given with the normal to the element’s plane and the local *x*-axis can be selected freely. The default orientation is parallel to the global XY plane with an angle smaller-equal than 90 degrees to the global *X*-axis. If the observer looks into the positive direction of the *z*-axis (thus from “above”), then they watch the nodes numbered counter-clockwise. If the element’s plane coincides with the global XY plane, the local and the global coordinate systems are then identical. Because the normal element remains plane, the bending and the membrane structural behaviour of the individual elements are decoupled. The element properties can thus be defined separately for both components. Additionally the consideration of the components of elastic support and a numerically conditional stiffness for the rotations around the shell normal occurs still. For a twisted element, the membrane and plate parts are generated by decoupling. Then they are coupled with each other via the twist of the element. Thus the element can represent curved shells very accurately.

**Figure 17:**
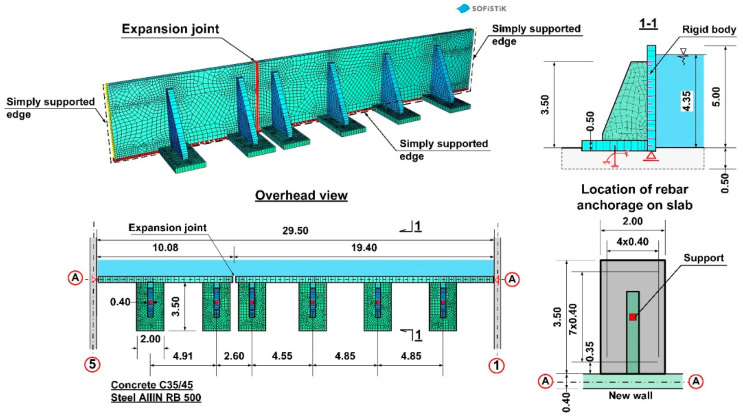
Numerical model for calculating wall support on axis A, which failed.

The ribs supporting the walls were designed as 40 cm thick reinforced concrete pilasters from C35/45 concrete. Due to considerable forces being transferred by the ribs to the foundation plate, additional support was planned in the form of a reinforced concrete base of 2.00 m × 3.50 m in the area and 0.50 m in height. The need to extend the base of the ribs resulted from insufficient flexural load-bearing of the existing foundation slab. While pressing on the slab, the rib generates flexural moments on the slab from ground resistance. Extending the base of the ribs allowed to distribute the loading over a larger area. What is more, the inclusion of a larger area of the slab secures additionally a twofold increase in height of the transverse cross-section for flexural and punching shear forces (50 cm + 50 cm = 100 cm). The basis for rib design was provided by several numerical models (FEM) which replicated the boundary conditions on the edges of the wall support. The numerical model was developed using quadrilateral mesh on surfaces (Figure 17). Additionally, rigid and non-linear elastic bonds were used. Articulated joints were used at the interface where the ribs connect to the wall. The external edges of the wall, which had been subjected to loading, were remodelled as articulated joints transferring only transverse forces. The analysis of the concrete wall according to the third-order theory, which contains non-linear analysis together with the analysis according to the second-order theory and the effects of the geometrical system modification, e.g., length modification for big deformations, was performed in SOFiSTiK [29]. The analysis of non-linear effects in SOFiSTiK was carried out by iterations using a modified Newton method with a constant stiffness matrix. The non-linear material behaviour of reinforced concrete in the shell elements was modelled by means a layer model with the arrangement of the crosswise reinforcement layers in their correct positions near the surfaces. The non-linear behaviour of the components of reinforced concrete was defined by non-linear uniaxial stress-strain laws of concrete (an increase of strength due to biaxial compressive behaviour is considered), consideration of tension softening of concrete after cracking dependent on fracture energy, trilinear stress-strain laws of reinforcing steel. Properties of the concrete material for a new structure were assumed as: a unit weight of 25 kN/m^3^, the Young’s Modulus of 34,077 N/mm^2^ (C35/45) and the Poisson’s ratio of 0.2, nominal strength 35 N/mm^2^, tensile strength 3.21 N/mm^2^. Properties of the concrete material for the old, existing structure were assumed as: a unit weight of 24 kN/m^3^, the Young’s Modulus of 31,476 N/mm^2^ (C25/30) and the Poisson’s ratio of 0.2, nominal strength 25 N/mm2, tensile strength 2.56 N/mm^2^. Properties of the reinforcing steel material properties were assumed as: the yield strength of N/mm^2^ and the Young’s Modulus of 200,000 N/mm^2^.

The SOFiSTiK [29] program makes it possible to study non-linear behaviour in such shells. An analysis according to the third-order theory, which contains non-linear analysis plus analysis according to the second-order theory and additionally the effects of the geometrical system modification, e.g., snap-through, length modification for big deformations and behaviour after buckling, was performed. Non-linear effects (e.g., plasticising, cracks) can be analysed only with iterations. This is done in Sofistik’s module ASE with a modified Newton method with a constant stiffness matrix. The advantages of the method are that the stiffness matrix does not have to be decomposed more than once and that the system matrix remains always positive. The line search method with the update of the tangential stiffness matrix is utilized for problems according to the second-order theory. The load increment is reduced here internally according to the available residual forces. If an iteration step proceeds into the right direction, i.e., in the direction of an energy minimum, then a new tangential stiffness that enhances the further iteration’s behaviour is generated, if necessary. Cracked elements are considered here also with reduced stiffness. The calculations were completed when the displacements agreed with the measured deflections during the tank leakage test (the differences between the actual displacements and the FEM values were within 5%). It was noted from the calculations that as a result of the wall cracking the stiffness of the shell elements was reduced by about 37%. For this reason, the reduced stiffness was assumed for the calculations of the wall together with the supports, reducing the modulus of elasticity of the wall by 37% (0.63 × *E*_ct_).

This approach was adopted to eliminate hidden cracks, about which the authors had no knowledge.

**Figure 18:**
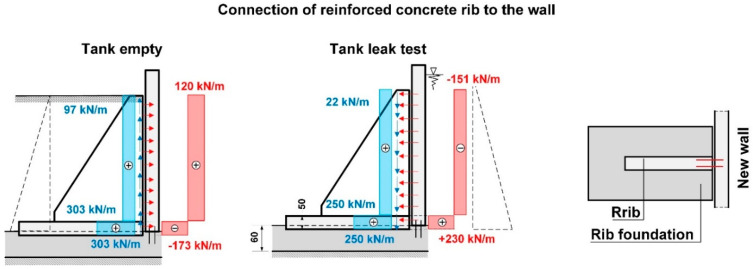
Design values for determining the wall-rib connection based on FEM calculations.

**Figure 19:**
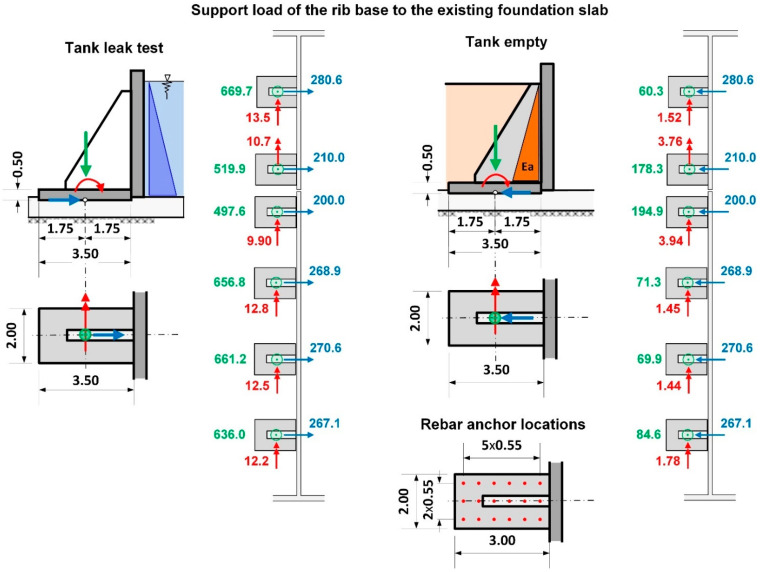
Reactions transferred to the anchor system based on FEM calculations.

The first stage of numerical calculation involved calculating the external forces in the ribs. The next step was to determine the transverse forces including the interactions at the interface of the rib–wall, side walls–wall, wall–foundation slab, and rib base–foundation slab. Determination of the forces provided the basis for designing the connection in the form of post-installed rebars using chemical adhesives (Figure 18 and Figure 19).

Based on a determination of the external forces in this way, the required strengthening of the pilasters was determined and the serviceability limit state (SLS) and ultimate limit state (ULS) were verified for the wall with the assumption that cracks would be no larger than 0.20 mm.

**Figure 20:**
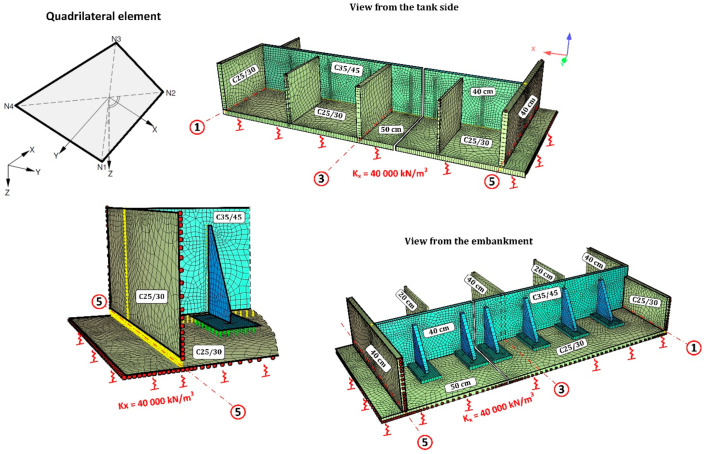
Sketch of the numerical model for verification of the flexural loading of the existing foundation slab.

**Figure 21:**
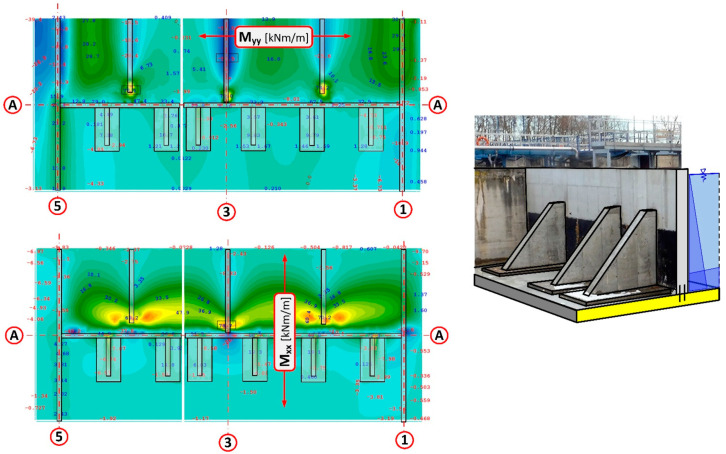
Values of the calculated moments for the foundation slab during the leak test together with the location of the walls and pilasters supporting the wall.

The last stage of the calculations was aimed at verifying the foundation slab in relation to reactions transferred by the base of the ribs to the underlying substrate. A corrected numerical model, based on the previous one, was developed for this purpose. The numerical model was extended to include the existing tank in order to approximate as closely as possible the existing field conditions, for instance: the Winkler model of the soil, the elastic half-space model, and the non-linear behaviour of cracked concrete. Meeting this goal required using the rigid linear connections to take into account the connections at the interface between the bottom of the rib base and the existing foundation slab. A sketch of the most important elements of the numerical model is presented in Figure 20, whereas the calculated values of the flexural moments in the foundation slab are presented in Figure 21.

**Figure 22:**
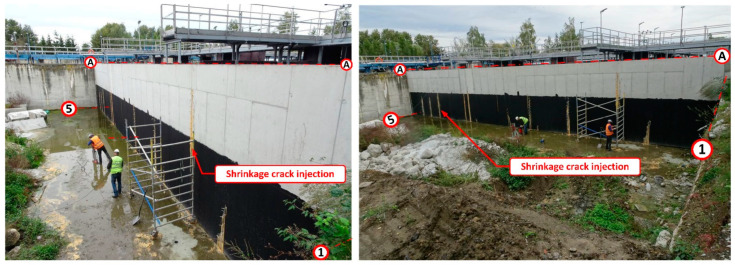
Injection in vertical cracks resulting from concrete shrinkage.

**Figure 23:**
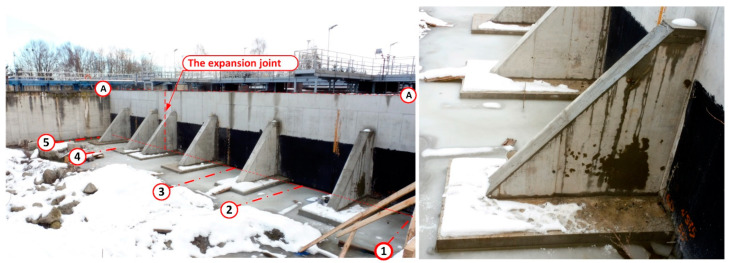
Strengthening of the wall of the biological reactor on the A axis with reinforced concrete supports.

**Figure 24:**
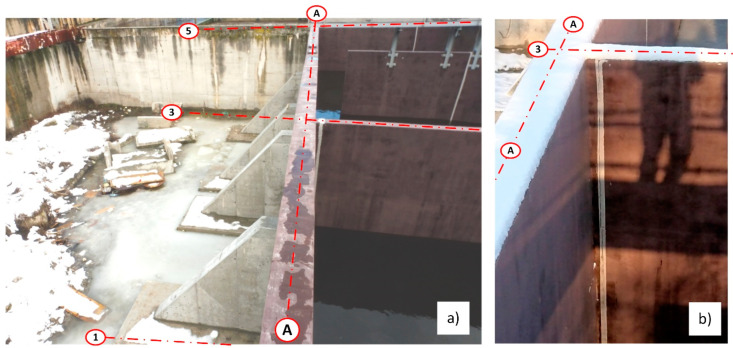
View of the strengthened wall of the bioreactor on axis A after the leak test, (**a**) view from the side of the supports; (**b**) view of the repaired expansion joint (dilatation) of the perpendicular wall in axis 3.

**Figure 25:**
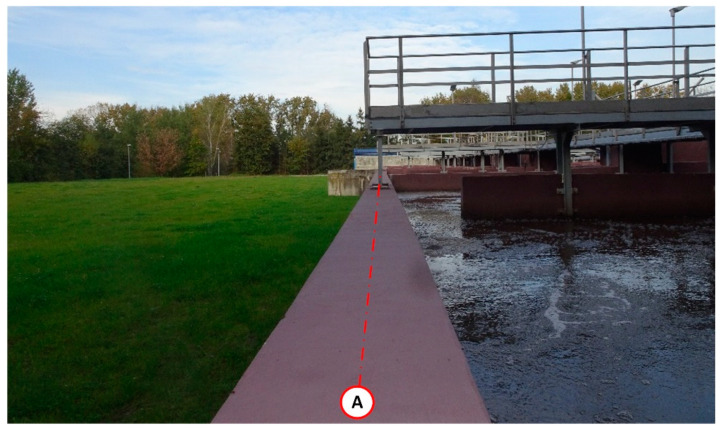
Wall view after repair.

Numerical analysis and static calculations carried out by the authors provided the basis for the preparation of a repair design. Prior to applying the pilasters, all of the shrinkage cracks, which appeared during the leak test, were sealed. The sealing was carried out by means of injection (Figure 22). The next step involved the renovation of the coatings of the tank interior and the installation of the bases for the pilasters. Finally, the reinforced concrete pilasters were installed and the insulation on the covering of the walls and supports from the ground side was restored (Figure 23). After completing all the construction works, a leak test was carried out again (Figure 24). It was successful. After the repair and leak test, the tank was covered with soil (Figure 25).

## 7. Discussion

This paper confirms that adopting the appropriate technical solution and verifying whether the assumed boundary conditions can be realised are an essential part of the design process. In the case discussed, the whole design process was directed, above all, to optimising the structure from the point of view of minimising the volume of concrete to be used and simplifying the implementation technology. The design engineer did not verify whether it was possible to anchor the wall in the foundation slab and, in consequence, adopted the length of the rebar strengthening based on experience in accordance with the nominal catalogue value. No verification was carried out using relevant calculations, as for example in [20]. The design project was approved, and implementation was initiated. Until the leak test, there were no signs of a problem with anchoring the new wall in the existing foundation slab. During the test, several problems occurred, for instance, displacement of the wall, damage to the extension joint, and as a result danger of collapse of the whole wall. The authors analysed the possibility of making an anchorage as was done. The result of these calculations confirmed that it was not possible to obtain a rigid connection to the foundation slab of the existing tank. It should be noted that the solution proposed would have been successful, providing that the water accumulation level was no greater than ~3.10 m and assuming safety factor *γ*_f_ = 1.50. It is also worth mentioning that the problem of the lack of anchoring would not have appeared if the leak test of the tank had been carried out after infilling unused space behind the wall (Polish regulations allow such practice). In such a situation, the loading from liquid pressure is balanced by a response from the ground. The problem might have revealed itself only during the next renovation when the tank would have been empty. The values of the anchorage moment with pressure from the ground are smaller and can withstand the water accumulation foreseen in the analysed situation of water surface at about 3.0 m, which would have suggested that the proposed solution might have succeeded. The problem with incorrect anchoring depth is a common design problem. The authors discuss all the significant problems that appear in relation to adopting such a solution and present how to make corrections in order to sensitise design engineers and building inspectors to the need to carefully verify these types of solution. All the significant aspects and problems related to the calculations which were carried out for other engineers and researchers to make use of the insights presented have been also included.

At the end of the considerations, the authors point out that the reason for the failure was the lack of knowledge about the anchoring rebar in the existing structure. The designer treated the rebar as a simply injectable adhesive anchor without considering the structural requirements and guidelines of the manufacturer of the anchoring system. The problem of the proper interpretation of construction conditions for deep anchoring of rebars requires good knowledge of PN-EN 1992-1-1 [31], considering this together with annexes such as DD CEN-TS 1992-4-5:2009 [18]. In many countries, these annexes have not yet been translated into their native languages. This might be the probable reason why the designers have not become familiar with them. The designers exclusively rely on the guidelines of anchor system manufacturers to uncritically adopt the dimensioning methods contained therein. However, it must be emphasised that, an approach derived from the classical methods results in repetitive and confirmed results. In the case of deep anchoring of reinforcing bars, the dimensioning methods vary as they are based on different assumptions. It follows that suppliers of anchoring systems should participate in the form of supervision during the design process and during construction to ensure that the solution is correctly adopted. Currently, on the market, the support for designers is limited to a calculation program that automatically selects the right anchor, leaving the designer without understanding the results. The last stage of the design process is on site. It is advisable for the manufacturers to prepare handy guidelines in the form of a simple procedures that would allow the designers to assess the correct length of anchorage of the bar or anchor by supervising the construction.

## 8. Conclusions

Strengthening the structure with inappropriate boundary conditions is a very common mistake. According to the foundation slab, a typical problem is to verify the wall anchorage only by checking the local failure mechanisms (Figure 14).

**Figure 26:**
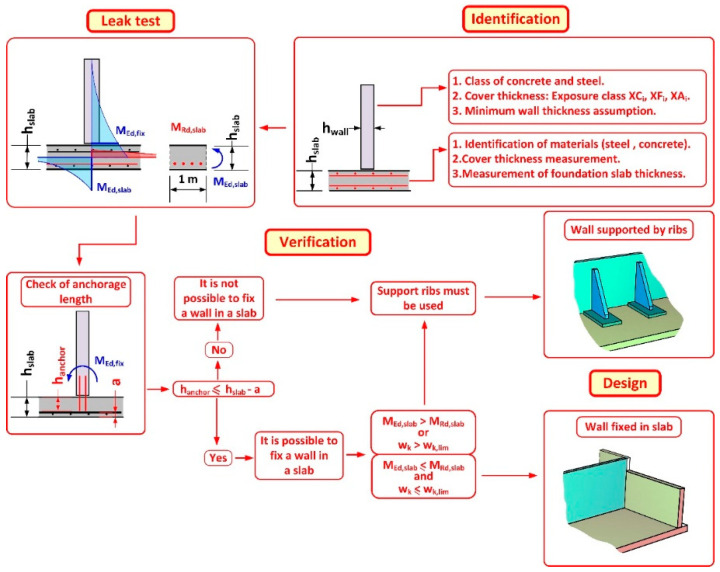
Proposed IVD (identification, verification, design) diagram for the design of new walls in existing reinforced concrete tanks.

It is also common practice to use simple computer programs prepared by anchor manufacturers. Furthermore, the examples with the anchorage design are prepared considering only the anchors and neglecting among other things the load-bearing capacity of the reinforced concrete node. This node will be formed after the wall is fixed in the existing foundation slab. The authors have presented the entire procedure using all the necessary equipment to assess the quality of the material, the distribution of the reinforcement, and the strength parameters of concrete. The authors proposed also the IVD (identification-verification-design) scheme (Figure 26) to make the design easier.

## Figures and Tables

**Table 1:** Results of compression tests on samples obtained from the foundation slab, subjected to cutting and grinding.

Number of Measurements	Compression Strength [MPa]			
	Mean	Range	Standard Deviation	Compliance with Criteria of Concrete Compression Strength in Accordance with PN-EN 13,791 [28]
9	33.7	30.7–36.1	1.7	*f*_ck,is_ = *f*_is,lowest_ + 4 = 30.7 + 4 = 34.7 MPa 34.7 MPa > 26 MPa for C25/30 *f*_ck,is_ = *f*_m(n),is_ − k = 33.7 − 6 = 27.7 MPa (for n = 9 ⇒ k = 6) 27.7 MPa > 26 MPa for C25/30

**Table 2:** Results of calculations of load-bearing capacity of the rebars in the foundation slab without consideration of shearing [18].

**Steel failure**															
$N_{Rk,s}=A_{s}\times f_{uk}=314{\;\mathrm{mm}}^{2}\times 550\frac{N}{{\mathrm{mm}}^{2}}=172.70\;\mathrm{kN}$															
N_Rk,s_			γ_Ms_			N_Rd,s_				N_Sd_—one anchor			β_N,s_		
172.7 kN			1.40			123.36 kN				80.06 kN			64.9%		
**Combined pull-out and concrete cone failure**															
$N_{Rk,p}=N_{Rk,p}^{0}\times \frac{A_{p,N}}{A_{p,N}^{0}}\times \Psi_{s,Np}\times \Psi_{g,Np}\times \Psi_{ec,Np}\times \Psi_{re,Np}$															
$N_{Rk,p}^{0}$	$A_{p,N}$			$A_{p,N}^{0}$			$\Psi_{s,Np}$		$\Psi_{g,Np}$			$\Psi_{ec,Np}$			$\Psi_{re,Np}$
128.18 kN	720,409 mm^2^			277,729 mm^2^			1.000		1.206			1.000			1.000
N_Rk,p_			γ_Mp_			N_Rd,p_				N_Sd_			β_N,p_		
400.99			1.50			267.33				560.39			**209.6%**		
**Concrete cone failure**															
$N_{Rk,c}=N_{Rk,c}^{0}\times \frac{A_{c,N}}{A_{c,N}^{0}}\times \Psi_{s,N}\times \Psi_{re,N}\times \Psi_{ec,N}$ $\tau_{Rk}=8.20\;{N/\mathrm{mm}}^{2}\;\;\tau_{Rk,\mathrm{ucr}}=13.00\;{N/\mathrm{mm}}^{2}\;\;\;\;\;\;\;k_{ucr}=7.20$															
$N_{Rk,c}^{0}$		$A_{c,N}$			$A_{c,N}^{0}$			$\Psi_{s,N}$			$\Psi_{re,N}$			$\Psi_{ec,N}$	
155.88 kN		119,2500 mm^2^			562,500 mm^2^			1.000			1.000			1.000	
N_Rk,c_			γ_Mc_			N_Rd,c_				N_Sd_			β_N,c_		
330.48			1.50			220.32				560.40			**254.3%**		

**Table 3:** Results of calculations of load-bearing capacity of anchorage of the rebars in the foundation slab with consideration of lateral force [18].

**Steel failure without lever arm**									
$V_{Rk,s}=0.5\times A_{s}\times f_{uk}=0.5\times 314{\;\mathrm{mm}}^{2}\times 550\frac{N}{{\mathrm{mm}}^{2}}=86.35\;\mathrm{kN}$									
V_Rk,s_		γMs		V_Rd,s_		V_Sd_—one anchor			β_V,s_
172.7 kN		1.50		123.36 kN		132 kN/14 = 80.06 kN			16.4%
**Concrete pry-out failure**									
$V_{Rk,cp}=k\times N_{Rk,c}=2\times N_{Rk,c}^{0}\times \frac{A_{c,N}}{A_{c,N}^{0}}\times \Psi_{s,N}\times \Psi_{re,N}\times \Psi_{ec,N}$ $\tau_{Rk}=8.20\;{N/\mathrm{mm}}^{2}\;\;\tau_{Rk,\mathrm{ucr}}=13.00\;{N/\mathrm{mm}}^{2}\;\;\;\;\;\;\;k_{ucr}=7.20$									
$N_{Rk,c}^{0}$	$A_{c,N}$		$A_{c,N}^{0}$		$\Psi_{s,N}$		$\Psi_{re,N}$		$\Psi_{ec,N}$
155.88 kN	1,669,500 mm^2^		562,500 mm^2^		1.000		1.000		1.000
V_Rk,cp_		γ_Mcp_		V_Rd,cp_		V_Sd_		β_V,cp_	
925.33 kN		1.50		616.89 kN		132.00 kN		21.4%	

**Table 4:** Degree of effort of anchorage in the foundation slab of the tank.

**Utilization of tension and shear**				
Steel failure β_N,s_	Combined pull-out and concrete cone failure β_N,p_	Concrete cone failure _N,c_	Steel failure without lever arm β_V,s_	Concrete pry-out failure β_V,cp_
64.9%	209.6%	254.4%	16.4%	21.4%
**Resistance to combined tensile and shear load**				
Steel utilisation				
$**\beta_{N,s}=0.65<1—$proof fulfilled $\beta_{V,s}=0.16<1—$proof fulfilled $\beta_{N,s}{}^{2}+\beta_{V,s}{}^{2}=0.45<1$—proof fulfilled				
Concrete utilisation				
$\beta_{N,c}=2.54\gg 1—$**proof not fulfilled** $\beta_{V,cp}=0.21<1—$proof fulfilled $\frac{\beta_{N,c}+\beta_{V,cp}}{1.20}=2.30\gg 1—$**proof not fulfilled**				

## Data Availability

Not applicable.
